# Upward Lightning at Wind Turbines: Risk Assessment From Larger‐Scale Meteorology

**DOI:** 10.1029/2023JD039505

**Published:** 2023-12-29

**Authors:** Isabell Stucke, Deborah Morgenstern, Gerhard Diendorfer, Georg J. Mayr, Hannes Pichler, Wolfgang Schulz, Thorsten Simon, Achim Zeileis

**Affiliations:** ^1^ Institute of Statistics University of Innsbruck Innsbruck Austria; ^2^ Institute of Atmospheric and Cryospheric Sciences University of Innsbruck Innsbruck Austria; ^3^ Department of ALDIS (Austrian Lightning Detection & Information System) OVE Service GmbH Austria Vienna; ^4^ Department of Mathematics University of Innsbruck Innsbruck Austria

**Keywords:** risk assessment of upward lightning, wind turbines, random forest, meteorological reanalysis, Gaisberg Tower, Saentis Tower

## Abstract

Upward lightning (UL) has become a major threat to the growing number of wind turbines producing renewable electricity. It can be much more destructive than downward lightning due to the large charge transfer involved in the discharge process. Ground‐truth lightning current measurements indicate that less than 50% of UL could be detected by lightning location systems (LLS). UL is expected to be the dominant lightning type during the cold season. However, current standards for assessing the risk of lightning at wind turbines mainly consider summer lightning, which is derived from LLS. This study assesses the risk of LLS‐detectable and LLS‐undetectable UL at wind turbines using direct UL measurements at instrumented towers. These are linked to meteorological data using random forests. The meteorological drivers for the absence/occurrence of UL are found from these models. In a second step, the results of the tower‐trained models are extended to a larger study area (central and northern Germany). The tower‐trained models for LLS‐detectable lightning are independently verified at wind turbine sites in this area and found to reliably diagnose this type of UL. Risk maps based on cold season case study events show that high probabilities in the study area coincide with actual UL flashes. This lends credibility to the application of the model to all UL types, increasing both risk and affected areas.

## Key Points

- Tower‐trained random forests can discriminate between high and low risk of upward lightning (UL) at wind turbines from larger‐scale meteorology
- Large values of convective precipitation, vertical velocity and ice crystals, together with convective available potential energy are most important for UL
- Regions with elevated terrain and coastlines perpendicular to the impinging wind field have an increased risk of UL

## 1 Introduction

The growing importance of renewable energy production has recently led to a significant increase in the number of wind turbines (e.g., Darwish & Al‐Dabbagh, 2020; Jørgensen & Holttinen, 2022). As these structures are typically taller than 100 m, the initiation of upward lightning (UL) propagating from the tall structure toward the clouds is facilitated (Berger, 1967). A tall structure is more likely to experience UL because it is exposed to a stronger electric field compared to the ground (e.g., Mazur, 2016). Structures shorter than 100 m mainly experience downward lightning (DL) with leaders propagating from the clouds toward the earth's surface (e.g., Rakov & Uman, 2003).

As wind turbines become taller, UL is the main weather‐related cause of severe damage to them (e.g., Matsui et al., 2020; Montanyà et al., 2016; Pineda et al., 2018; Rachidi et al., 2008; Zhang & Zhang, 2020). It can be critically destructive because its initial continuous current (ICC) may last up to several hundreds of milliseconds and transfer large amounts of charge (e.g., Diendorfer et al., 2011; Rakov & Uman, 2003; Saba et al., 2016). Ground‐truth lightning current measurements on the specially instrumented tower at the top of the Gaisberg mountain (Austria, Salzburg) show that more than 50% of UL is not detected by conventional lightning location systems (LLS). While LLS can detect ICC‐return strokes and many ICC‐pulses due to their short current rise times responsible for a sufficiently strong electromagnetic field, they cannot detect a certain subtype of UL with only an ICC (Diendorfer et al., 2015; March et al., 2016). Although there are towers providing ground‐truth lightning current data for LLS‐detected UL, such as the Säntis Tower in Switzerland, the Gaisberg Tower is the only instrumented tower in Europe providing full information on the occurrence of both LLS‐detected and LLS‐undetected UL.

International standards for lightning protection of wind turbines (IEC 61400‐24, 2019) might crucially underestimate the occurrence of UL at wind turbines as they currently rely on only three factors: The height of the wind turbine, the local annual flash density derived from LLS, and an environmental term that includes factors such as terrain complexity or altitude (Becerra et al., 2018; March, 2018; Pineda et al., 2018; Rachidi et al., 2008). Summer lightning activity clearly dominates the annual local flash density due to large amounts of DL caused by deep convection (see Supporting Information S1). However, UL is expected to be the dominant lightning type at wind turbines with a tendency to be even more important in the colder season (Diendorfer, 2020; Rachidi et al., 2008). Furthermore, the risk assessment standards cannot take into account LLS‐undetected UL, but only LLS‐detected UL if a tall structure is present.

The main objective of this study is to assess the risk of LLS‐detected UL and LLS‐undetected UL on wind turbines over a larger area. Although LLS are available to analyze LLS‐detected UL at tall structures, direct lightning current measurements show that a significant proportion is missed. Recognizing that conventional LLS cannot assess the full risk of UL at wind turbines, a new approach is used in this study.

Since the occurrence of UL can only be provided by ground‐truth lightning current measurements, the present study uses machine learning techniques to link the occurrence of UL to the larger‐scale meteorological environment. These techniques form the basis for building and training the statistical models that will ultimately be used to assess the risk of UL over an entire study area. Specifically, this study uses conditional inference random forests (Hothorn & Zeileis, 2015), which account for the highly non‐linear and complex interactions between the incidence of UL on the tall structures and the atmosphere. Several steps are required to achieve the main goal.

From direct lightning current measurement data at two instrumented towers in Austria (Gaisberg Tower) and Switzerland (Säntis Tower), two models are constructed: One for LLS‐detected UL and one for both LLS‐detected and LLS‐undetected UL (LLS‐detected + LLS‐undetected UL). The aim of these models is, first, to determine whether there is a relationship between larger‐scale meteorological variables and the occurrence of UL and, second, to demonstrate how well larger‐scale meteorology can serve as a diagnostic tool for inferring the occurrence of UL.

The advantage of the availability of LLS‐detected UL data helps to verify whether the results from the instrumented towers are transferable. The idea is to extract wind turbine sites within the study area and identify all lightning strikes to them from the colder season (ONDJFMA) using LLS data. Success in reliably identifying LLS‐detected UL from larger‐scale meteorology in combination with UL ground‐truth lightning current measurements provides greater confidence in the results when, in a final step, the risk of LLS‐undetected UL, which cannot be verified using LLS data, is assessed.

The following sections are organized as follows. Section 2 introduces the two instrumented towers that provide the necessary ground‐truth data for this study. The first is the Gaisberg Tower, which provides both LLS‐detected UL and LLS‐undetected UL, and the second is the Säntis Tower, which provides only LLS‐detected UL. Furthermore, this section presents the identification of lightning at wind turbines in the study area and the meteorological data used. Section 3 summarizes the procedures and main results from the two instrumented towers. Section 3.1 describes the basic principle of building a random forest model. Section 3.2 presents the performance of the models on the instrumented towers. Furthermore, the most important larger‐scale meteorological variables leading to a higher risk of UL are introduced (Section 3.3). Then, Section 4 presents the results of extending the models from the instrumented towers to the larger study area to find regions with a higher risk of experiencing UL. Section 4.1 investigates LLS‐detected UL on wind turbines and presents case studies. Section 4.2 then illustrates and discusses the risk of LLS‐detected UL and LLS‐detected + LLS‐undetected UL on wind turbines for the entire study period. Section 5 concludes and summarizes the most important findings.

## 2 Data

This study combines five different data sources: UL data measured directly at the Gaisberg Tower in Austria (Diendorfer et al., 2009) and at the Säntis Tower in Switzerland (Romero et al., 2012); LLS data measured remotely by the European Cooperation for Lightning Detection (EUCLID, Schulz et al., 2016); larger‐scale meteorological variables from the reanalysis database ERA5 (Hersbach et al., 2020); wind turbine locations identified using the ⓒ OpenStreetMap (OpenStreetMap contributors, 2020) database.

### 2.1 Direct UL Measurements at Instrumented Towers

**Figure 1:**
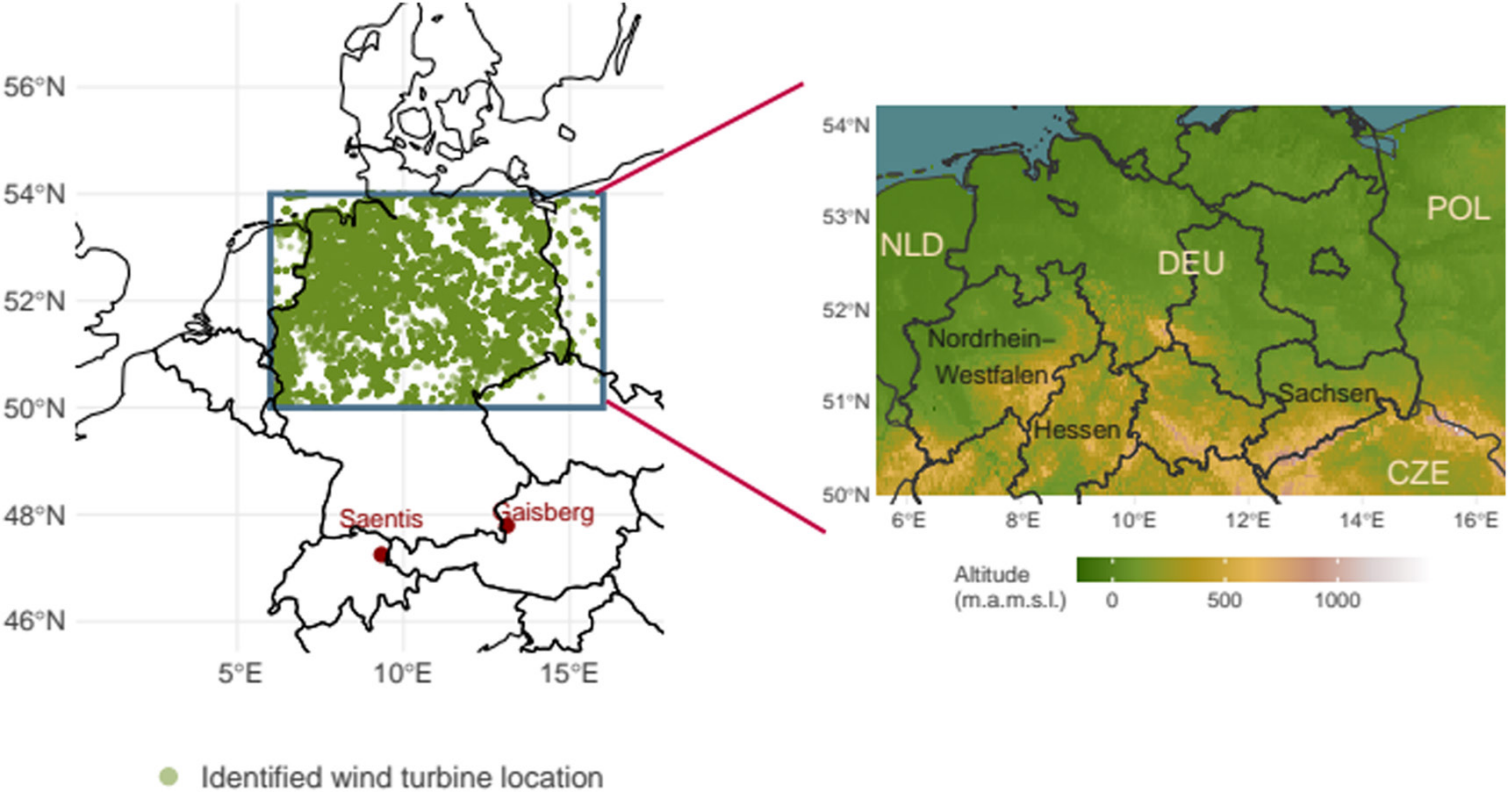
Geographic overview of the instrumented tower locations (Gaisberg and Säntis) as well as the study domain (box). Green dots are manually identified wind turbine locations based on ⓒ OpenStreetMap (2020). Right: topographic map of study domain showing altitude above mean sea level. Data taken from Shuttle Radar Topography Mission (Farr & Kobrick, 2000).

Figure 1 shows two of the very few instrumented towers for direct measurement of currents from UL. These are the Gaisberg Tower (1,288 m amsl, 47°48′N, 13°60′E) and the Säntis Tower (2,502 m amsl, 47°14′N, 9°20′E). Lightning at the instrumented towers is almost exclusively UL (Diendorfer et al., 2011). Gaisberg Tower recorded a total of 819 UL flashes between 2000 and 2015. Säntis Tower recorded 692 UL flashes between 2010 and 2017.

A sensitive shunt type sensor at Gaisberg allows measurement of all types of upward flashes regardless of the current waveform, that is, LLS‐detected UL and LLS‐undetected UL. However, the inductive sensors used by Säntis cannot measure upward flashes with only an ICC (about 50%, Diendorfer et al., 2015).

Direct UL current measurements are fundamental and critical to the construction or training and testing of the random forest models, which are extended to the larger study area after training on the tower data. The combination of data from both towers provides a sufficiently large data set and allows to build two types of models to study both LLS‐detected UL and LLS‐detected + LLS‐undetected UL.

### 2.2 LLS‐Detected UL at Wind Turbines and Study Domain

While the model training and testing takes place exclusively at Gaisberg and Säntis Tower, remotely detected lightning data from LLS EUCLID and wind turbine locations derived from ⓒ OpenStreetMap serve as verification approach of the statistical models assessing the risk of LLS‐detected UL for the selected study area.

Within the study area of 50°N–54°N and 6°E−16°E, 27,814 wind turbines have been installed by the end of 2020 (Figure 1). Due to its destructive potential and its severe underestimation in the international lightning protection standard (IEC 61400‐24, 2019, e.g., March, 2018), UL, and in particular the risk of UL at wind turbines, shall be explicitly considered in this study. After extracting the exact locations of wind turbines in the study domain, lightning strikes within a 0.003° circular area (approximately within 300 m radius) detected by EUCLID are identified and assumed to be UL. This radius was chosen to be large enough so that all UL flashes from a single wind turbine would be within this 300 m area, due to the limited average location accuracy of EUCLID of about 100–250 m (Schulz et al., 2016). In addition, it was chosen to be small enough to avoid overlap when turbines are closely spaced in wind farms. EUCLID cannot distinguish between UL and DL, so there is a possibility that the latter could be misclassified as UL. To reduce this possibility, only the months from October to April, starting from October 2018 to December 2020, are considered in the verification part of the study. As UL is expected to be dominant in the colder season compared to DL (e.g., Saito et al., 2015; Yuan et al., 2017), the proportion of potential misclassifications should be negligible. An additional check with data from the Austrian Lightning Detection and Information System between 2000 and 2015 found not a single DL flash within a 300 m radius around the Gaisberg Tower when there was no UL flash at the Gaisberg Tower in the same hour.

Note that EUCLID detects DL with an efficiency of more than 90% (Schulz et al., 2016), but UL only with less than 50% (Diendorfer et al., 2015).

### 2.3 Meteorological Data

Meteorological data are critical both for training and testing the models at the two instrumented towers to explain UL, and for extending the models to the study area with the prevailing meteorological environment.

ERA5 provides an hourly reanalysis of the state of the atmosphere. It has a resolution of 31 km horizontally (grid cell size of 0.25° × 0.25°) and 137 levels vertically. This study uses 35 directly available and derived surface, model level, and vertically integrated variables. These reflect variables relevant to cloud electrification, lightning, and thunderstorms (Morgenstern et al., 2022). A complete list of variables can be found in Supporting Information S1. The data are spatially and temporally bilinearly interpolated to each Gaisberg and Säntis Tower UL observation that occurred from 2000 to 2015 (Gaisberg) and from 2010 to 2017 (Säntis), as well as to each grid cell within the study area for which reanalysis data from 2018 to 2020 are used.

## 3 Methodological Procedures and Findings From the Instrumented Towers

This section provides the necessary background information on the basic methods as well as important results from the analysis of the instrumented Gaisberg Tower and Säntis Tower. Three different aspects will be covered: First, the principle of how the basic model, a random forest, is trained and tested. Second, the performance of the models at the tower(s) and third, which variables are most important to identify favorable larger‐scale meteorological conditions for UL to occur at the tower(s) or not.

### 3.1 Training and Testing at the Instrumented Towers

A machine learning technique that has recently been widely applied in various scientific fields is used to link larger‐scale meteorology and the occurrence of UL at the instrumented towers. A previous study by the same authors shows the successful application of random forest models to investigate the triggering mode and flash type of UL (Stucke et al., 2023). Random forests (Breiman et al., 1984) are highly flexible and able to handle nonlinear effects, capturing complex interactions with respect to the stated modeling problem (Strobl et al., 2009).

The occurrence versus non‐occurrence of UL is a binary classification problem, which is tackled using 35 larger‐scale meteorological variables (predictors or “features”). Each meteorological predictor is linked to the response (“labels”), that is, a situation with or without UL at Gaisberg or Säntis Tower using a random forest. A random forest combines predictions from multiple decision trees trained on randomly selected subsamples of the input data.

Specifically, the trees in the random forest are constructed by capturing the association between the binary response and each of the predictor variables using permutation tests (also known as conditional inference, see Strasser & Weber, 1999). The idea is that at each step in the recursive tree construction, the one predictor variable that has the highest (most significant) association with the response variable is selected. Then, the data set is split with respect to this predictor variable in order to separate the different response classes as well as possible. The splitting is repeated recursively in each of the subsets of the data until some stopping criterion (e.g., regarding significance or subsample size) is met. The forest combines 500 of such trees, where each tree is learned on randomly subsampled two‐thirds of the full data set, and only six randomly selected predictors are considered in each split. Finally, the random forest averages the predictions from the ensemble of trees, which stabilizes and improves the prediction performance. See Hothorn et al. (2006) and Hothorn and Zeileis (2015) for more details on the algorithm and implementation.

The input model response, that is, does UL occur or not, is sampled so that the two classes are balanced, that is, situations with and without UL are present in equal proportions. Since UL is rare, omitting this procedure, might lead to unbalanced results and potentially incorrect conclusions. The models compute the socalled conditional probability on the data not considered in the training of the models, that is, on the omitted day. We call the probability “conditional” because of the balanced model response setup. In order to compute the conditional probability of UL also on days without UL, days without UL are randomly sampled from each season between 2000 and 2017.

To be able to validate the resulting tower‐trained models, the input data are split into training and test data samples. The training data are used to train the models, and the unseen test data are used to evaluate the ability to correctly identify or “diagnose” whether UL occurs or not. Therefore, high computed conditional probabilities when UL occurred at Gaisberg or Säntis in the particular situation (i.e., a high true positive rate) and low conditional probabilities when no UL occurred (i.e., a low false positive rate), refer to a high socalled diagnostic ability, hence to a high performance of the random forest models.

After splitting the input model response into training and test data samples, leave‐one‐out cross‐validation is used to test the models for LLS‐detected UL and LLS‐detected + LLS‐undetected UL. The first model for LLS‐detected UL uses both Säntis data and Gaisberg data to increase the size of the training data. The particular flash type that cannot be detected by the Säntis Tower is omitted from the Gaisberg data during training to ensure consistency. The second model for LLS‐detected + LLS‐undetected UL uses only Gaisberg data because only the Gaisberg Tower provides complete information on all subtypes of UL. Between 2000 and 2015, the Gaisberg Tower experienced 247 unique days with UL flashes. Between 2010 and 2017, the Säntis Tower experienced 186 unique days. Combining the UL days from both towers yields 406 unique days with UL. Each training input data set omits one of the 247 (406) days with UL to use it as test data. This is repeated until each of the 247 (406) days is omitted once for training the random forest models. This results in 247 (406) different models trained on situations with and without UL. The reason for using many different random forest models is to represent as many different meteorological combinations and scenarios as possible, since each training data set contains randomly sampled situations without UL, which can vary widely throughout the year. Furthermore, each random forest model is by definition slightly different from the other random forest model, as each model introduces different sources of randomness during construction (Hothorn & Zeileis, 2015; Hothorn et al., 2006).

### 3.2 Diagnostic Ability of the Tower‐Trained Random Forests

**Figure 2:**
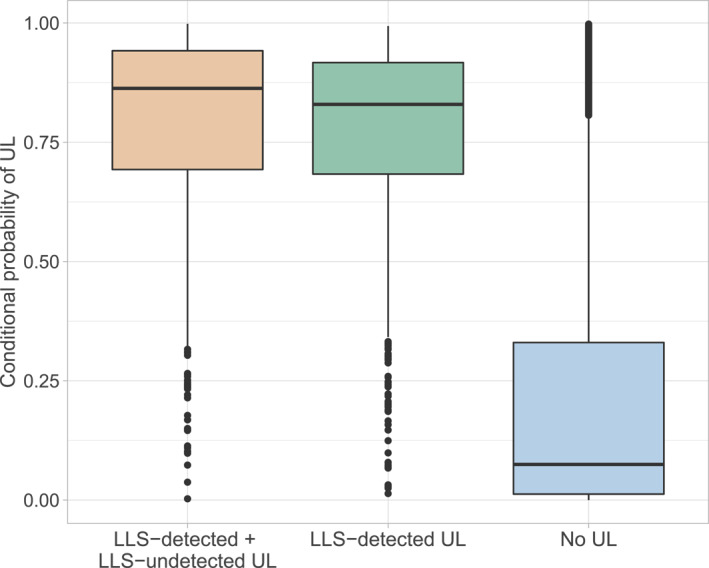
Distributions of computed conditional probabilities in situations with or without Upward lightning (UL) flashes. Boxplots show the median (vertical middle line), the interquartile range (IQR, 25th to 75th percentile), and whiskers (±1.5 × IQR). The black dots indicate the outliers falling outside the whiskers. (Left) conditional UL probability given that UL was observed in the particular minute (true positive) based on Gaisberg data including all subtypes of UL. (Center) conditional UL probability given that UL was observed in the particular minute based on Gaisberg and Säntis data combined. (Right) conditional UL probability on randomly sampled days without UL flashes (false positive).

The tower‐trained random forest models can reliably diagnose both LLS‐detected UL and LLS‐detected + LLS‐undetected UL when tested on unseen withheld data from the towers. Figure 2 summarizes the cross‐validated diagnostic ability according to the random forests for LLS‐detected + LLS‐undetected UL (Gaisberg) and LLS‐detected UL (Gaisberg + Säntis). The closer the conditional probability to a value of one, given that UL occurred, the higher the diagnostic ability. Both model ensembles show a similarly good diagnostic ability. The computed median conditional probabilities are about 0.8 that UL was observed in the respective situation (minute). Random forest models based on Säntis observations yield a median conditional probability of about 0.75 (not shown). These results indicate a high true positive rate. Similarly, for situations without UL (right), the conditional probabilities are low, indicating a low false positive rate.

The deviation among the median probabilities might stem from the difference in the ability of larger‐scale meteorology alone to perfectly model the UL occurrence. Other environmental factors might also influence the UL initiation at the two different geographical locations of the instrumented towers (e.g., March, 2018).

The fact that the random forest including LLS‐undetected UL has the highest diagnostic ability shows that the fraction not detected by conventional LLS might be largely represented by larger‐scale meteorology. This supports the idea to also investigate the risk of undetectable LLS‐undetected UL and not only LLS‐detected UL.

### 3.3 Meteorological Drivers for LLS‐Detected UL at the Instrumented Towers

**Figure 3:**
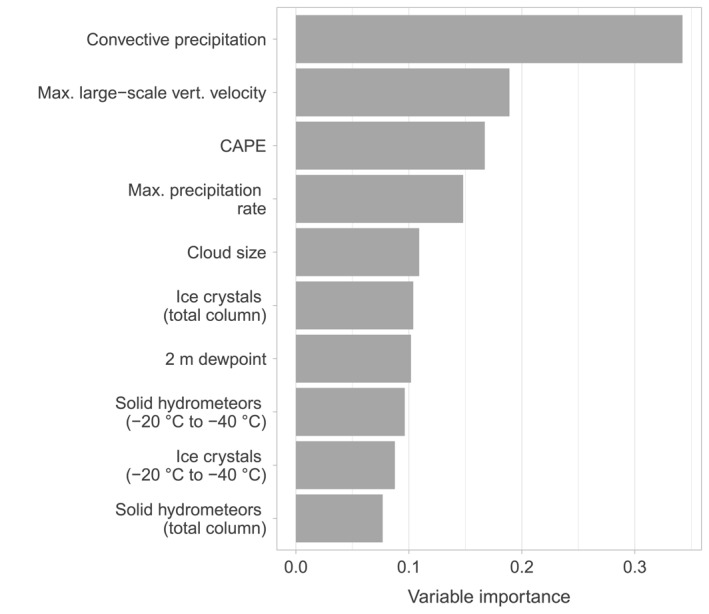
Median permutation variable importance according to 100 different random forests based on balanced proportions of situations with and without Upward lightning at the Gaisberg and Säntis Tower.

Random forests allow to assess the influence of individual variables on the diagnostic ability of the models. This is done by calculating the so‐called permutation variable importance. The idea is to break the relationship between the response variable and a predictor variable by neglecting its information when assessing the diagnostic ability of the models. Neglecting the information of a predictor variable is done by permutation, that is, randomly shuffling its values and then assessing how much the diagnostic ability decreases. Often, the forest will replace the neglected variable with one closely related to it, for example, convective precipitation with the maximum precipitation rate. Figure 3 visualizes the calculated median permutation variable importance according to 100 different random forest models for LLS‐detected UL. Each variable contributes to the performance of the model, but only the top 10 are shown. The variable importance numbers are unitless and are intended to reflect the importance of one variable relative to the rest. Each of the 100 models is based on a balanced proportion of situations with UL and randomly selected situations without UL to represent a multitude of different combinations of UL and no‐UL situations. The results for the LLS‐detected UL and LLS‐detected + LLS‐undetected UL models are very similar.

With a median value of 0.34, convective precipitation has the largest influence on the occurrence of UL according to the random forests based on direct observations from Gaisberg and Säntis Tower (Figure 3). Neglecting the information of this driver variable reduces the diagnostic ability the most. The second and third most important variables are the maximum larger‐scale vertical velocity (0.19) and the convective available potential energy (CAPE, 0.17). A statistical summary of the three most important variables shows that CAPE at both the Säntis Tower and the Gaisberg Tower is rather low when UL occurs (Gaisberg: median value of 68 J kg^−1^, Säntis: median value of 85 J kg^−1^). At the Gaisberg Tower, convective precipitation comes with a median of 0.38 mm (Säntis: 0.44 mm) and a maximum larger‐scale vertical velocity with a median of −1.5 Pa s^−1^ (Säntis: −1.0 Pa s^−1^). Other important variables are the maximum precipitation rate since previous postprocessing, the vertical size of the thundercloud, the amount of ice crystals and solid hydrometeors, and the 2 m dew point temperature.

Figure 3 reflects both the importance of convective precipitation and variables related to cloud physics such as particles in different phases, but also the importance of the wind field represented by the maximum larger‐scale vertical velocity, which is in line with (Stucke et al., 2022) and other studies showing the importance of wind for UL (e.g., Mostajabi et al., 2018; Pineda et al., 2019; Yuan et al., 2017).

The top three variables generally play the key role in any convective process. While no physical interpretation of the effect of the different variables on the UL is possible from the random forests alone, an exploratory approach should provide further insights.

**Figure 4:**
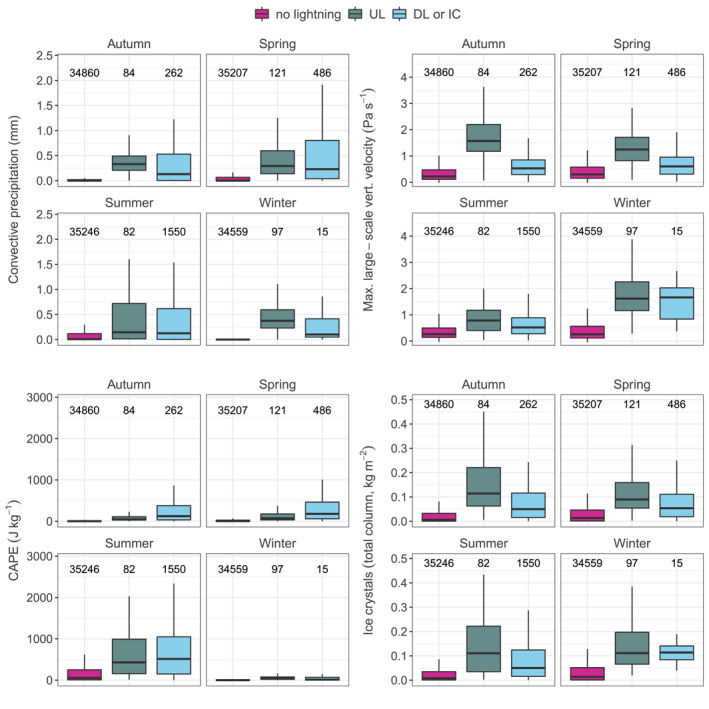
Distributions of convective precipitation, maximum larger‐scale vertical velocity, convective available potential energy, and total column ice crystals separated into autumn (SON), spring (MAM), summer (JJA), and winter (DJF) according to three scenarios: no lightning 15 km around the Gaisberg Tower (pink), Upward lightning at the Gaisberg Tower (dark gray) and downward lightning or intracloud lightning (IC) around the Gaisberg Tower (15 km, light blue). Numbers above the boxplots indicate number of observations according to the season and scenario between 2000 and 2015. Interpretation of boxplots same as in Figure 2 without outliers.

Figure 4 shows the seasonal variation of convective precipitation, the larger‐scale vertical velocity, CAPE and the total column ice crystal content according to three scenarios: no lightning around the Gaisberg Tower, that is, the seasonal “average,” UL at the Gaisberg Tower and lightning around the Gaisberg Tower (15 km) including DL and intra‐cloud lightning (IC) between 2000 and 2015. In both UL and DL or IC situations, all four variables are higher in median than the seasonal average, respectively. However, UL seems to occur with more convective precipitation, especially in winter and autumn, with stronger maximum larger‐scale vertical velocities throughout the year, and with a higher total column ice crystal content, especially in the transition seasons and in summer. This indicates that UL lightning requires enhanced cloud physical processes, such as large particle loadings and strong precipitation, in combination with an enhanced vertical (and horizontal) wind field (see also Stucke et al., 2022). These processes are even stronger for UL than for any other type of lightning, regardless of the season. DL and IC, on the other hand, have higher CAPE values throughout the year.

## 4 UL at Wind Turbines

**Figure 5:**
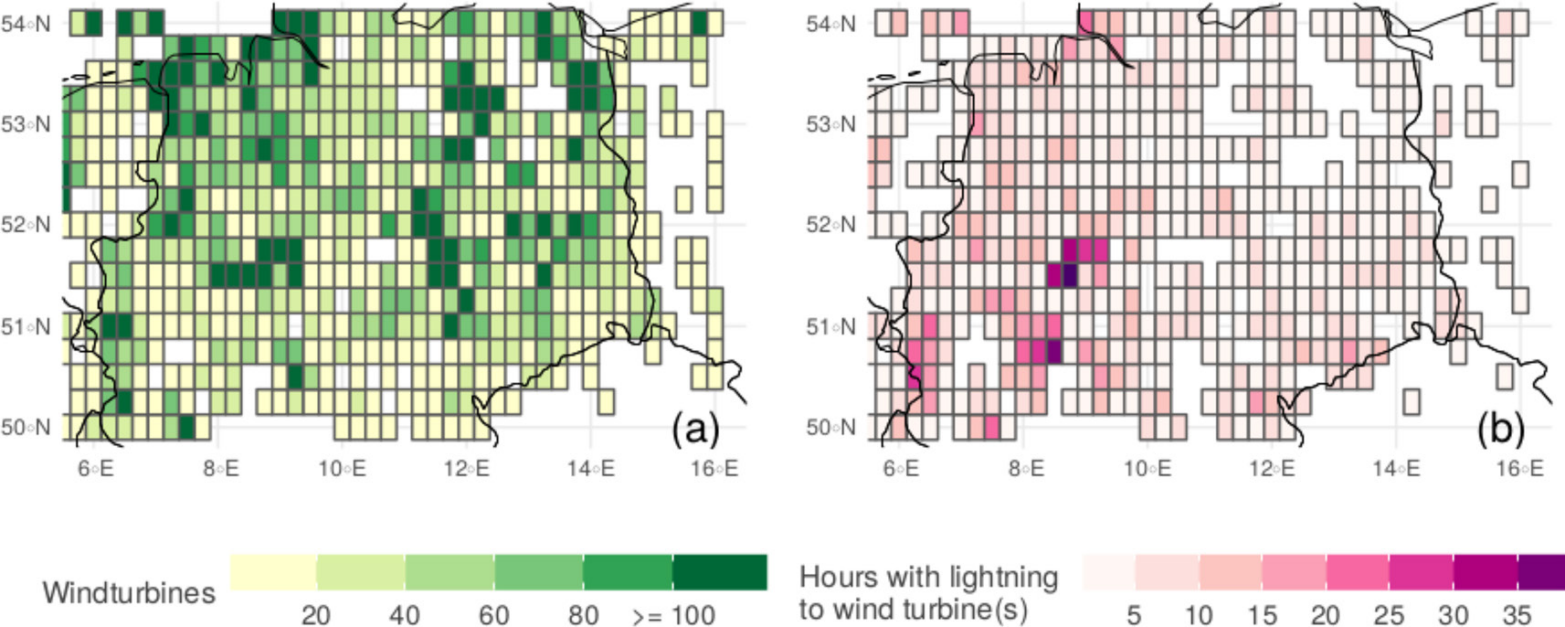
Panel (a) number of wind turbines per grid cell derived from ⓒ OpenStreetMap (2020) data. Panel (b) number of hours per grid cell with lightning at wind turbines derived from EUCLID data.

Extraction of wind turbine locations and identification of lightning strikes to them within 300 m in the cold season (ONDJFMA) shows that there are regions within the study area that experience UL more frequently than others (see Figure 5). Interestingly, the regions that experience UL more frequently (panel b, dark pink) coincide with regions with many wind turbines. In general, however, it can be observed that regions with a high number of wind turbines (panel a, dark green) do not necessarily coincide with a high number of UL flashes, as can be seen for example, in the northeastern parts of the study area. The following sections present and discuss the results of extending the results from the instrumented towers to the study area by extracting the locations of wind turbines and analyzing the lightning activity to them.

### 4.1 Identifying LLS‐Detected UL at Wind Turbines From Larger‐Scale Meteorological Conditions

The random forest models for LLS‐detected UL and LLS‐detected + LLS‐undetected UL, based on data from the two instrumented towers, identified larger‐scale meteorological variables that are the most important discriminators between situations with and without UL. The tower‐trained random forest models are now applied to the larger study area to assess the risk of UL at wind turbines from larger‐scale meteorological variables alone. Lightning measurements from LLS data will verify the results at identified wind turbine sites.

The following results are based on a similar procedure as described in Section 3.2, except that each grid cell (31 km × 31 km) of the study domain is used as test data instead of the cross‐validated data from the instrumented towers.

To increase the robustness of the results, again 100 different random forest models are used to compute the conditional probability of UL on the selected case studies over the study domain. The results in this section visualize the median conditional probabilities computed by the random forest models.

#### 4.1.1 Case Studies: LLS‐Detected UL at Wind Turbines

To illustrate the diagnostic ability of the tower‐trained random forests for LLS‐detected UL on days with UL flashes at wind turbines, three different case study days are selected from the colder seasons between 2018 and 2020 in the study area.

The selected case study days are characterized by typical weather situations for the colder seasons in the mid‐latitudes. The atmosphere in the transitional seasons and in winter tends to be highly variable and influenced by the succession of cyclones and anticyclones that determine the meteorological setting (Perry, 1987). In particular, the development and progression of mid‐latitude cyclones provide favorable conditions for so‐called wind field thunderstorms (Morgenstern et al., 2022). This type of thunderstorm is associated with, among other things, strong maximum larger‐scale vertical velocities, high precipitation amounts, and low but present CAPE.

**Figure 6:**
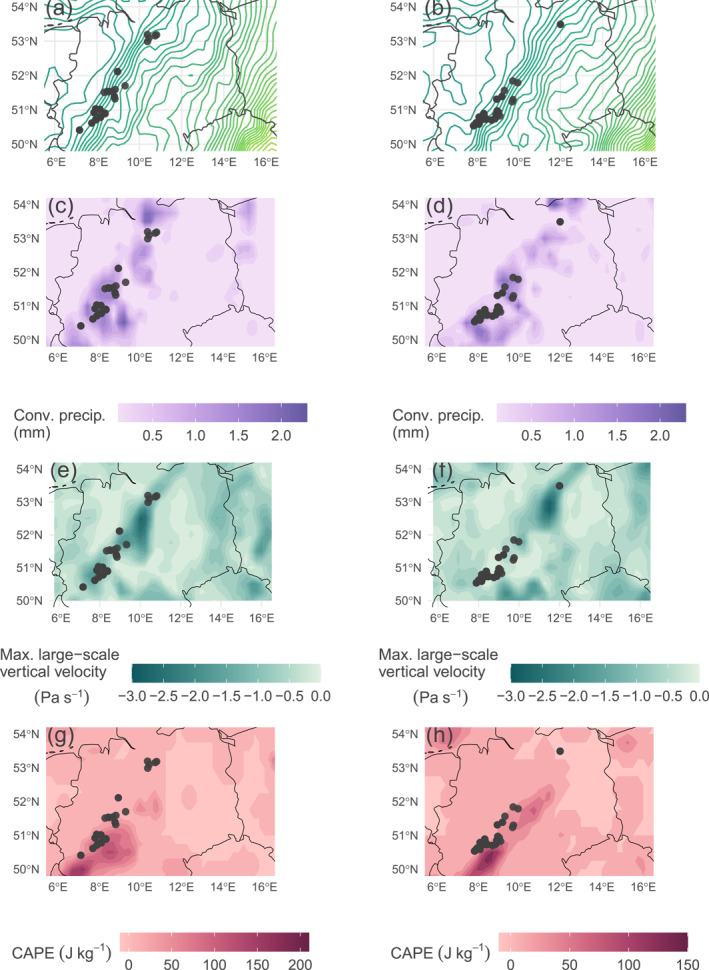
Larger‐scale meteorological setting on the 4 March 2019 over the study domain. Left column illustrates the setting at 13 UTC, right column at 14 UTC. From upper to lower: spatial distributions of isolines of the 850 hPa temperature (in intervals of 1 K), convective precipitation, the maximum larger‐scale vertical velocity (negative values is upward motion) and convective available potential energy. Darker colors indicates higher magnitude. Dark gray dots in all figures are flashes within the considered hour and ERA5 grid cell derived from lightning location systems EUCLID data.

The first case study is considered in more detail with respect to the drivers identified at the instrumented towers (Figure 3). Figure 6 illustrates the larger‐scale isotherm locations, spatial distribution of convective precipitation, maximum larger‐scale vertical velocity, and CAPE on 4 March 2019 at 13 UTC and 14 UTC. LLS detected lightning flashes at the identified wind turbines within the respective hour are indicated as dark gray dots.

The meteorological setting is determined by the passage of a cold front ahead of a trough around noon. Densely packed isotherms at 850 hPa crossing northern and central Germany from west to east indicate the approximate location of the cold front in panels a and b. The cold front implies locally increased amounts of convective precipitation in c and d, strong larger‐scale vertical velocities indicated by large negative values in e and f, and slightly increased but generally low CAPE in g and h compared to deep convection in summer. All three variables show maximally increased values in slightly different areas within the study area induced by the cold front. Convective precipitation shows increased values along the cold front, while the other two variables have locally more concentrated areas with maximum values (e.g., maximum larger‐scale vertical velocity in North/Central Germany).

**Figure 7:**
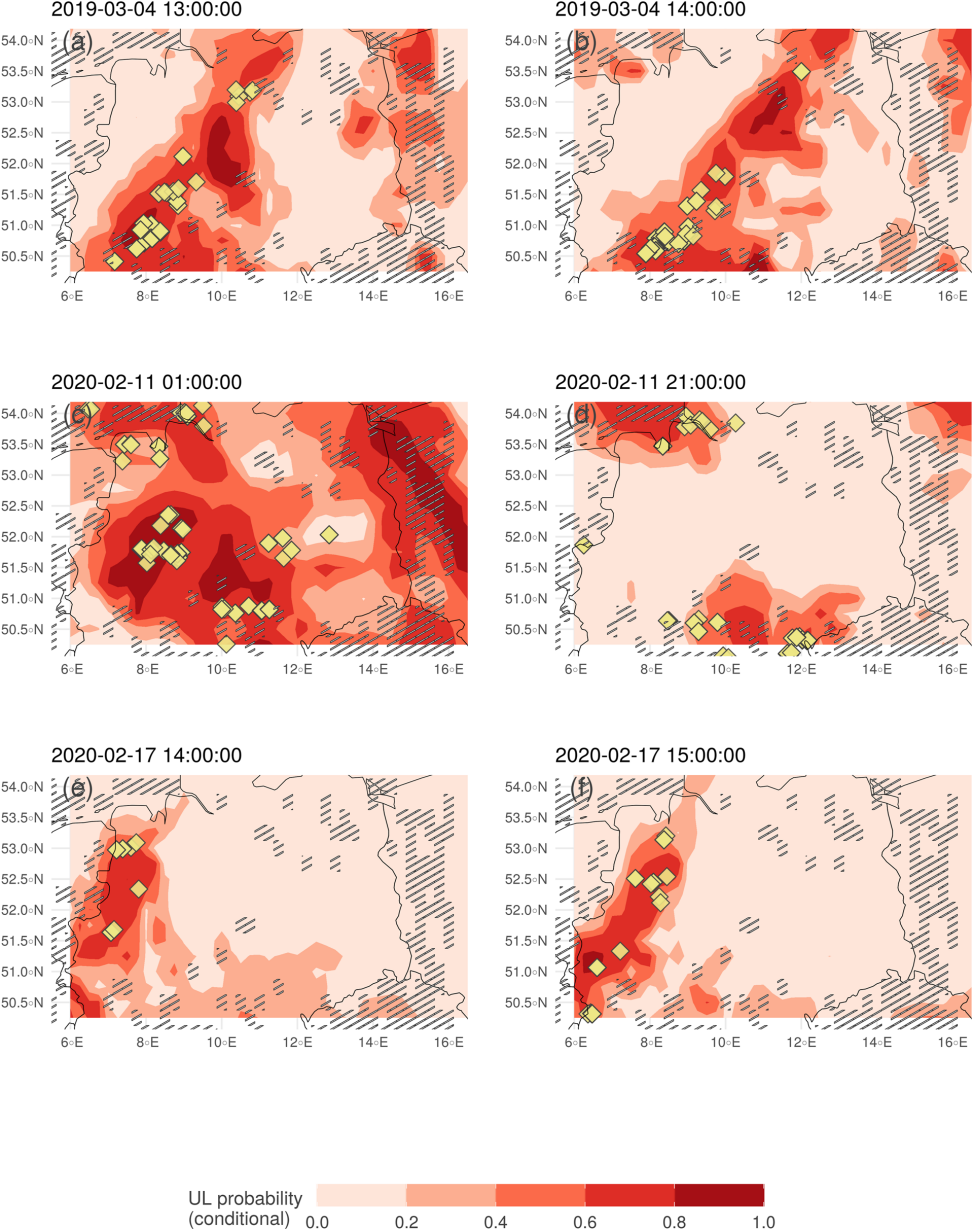
Median computed conditional probability of Upward lightning according to 100 random forest models based on Gaisberg and Säntis Tower data (red areas). Yellow symbols are flashes within the considered hour derived from EUCLID data. Gray shaded areas are grid cells without wind turbines.

Figure 7 visualizes the conditional probability computed by the random forest models in red colors for all three case study days. Panels a and b show the results for the particular case study discussed in Figure 6. The computed pattern is a result of combining the influence of the three driver variables. This suggests that no single variable can be responsible for the resulting probability map, but rather an interaction of different influencing variables resulting in areas of increased risk of experiencing UL.

The yellow symbols show lightning strikes over the hour considered. Identified lightning flashes in yellow require a wind turbine within a maximum distance of 300 m as described in Section 2. All other tall structures that may have experienced UL are not considered in this figure. Since the computed probabilities do not depend on wind turbine locations, high probabilities may result even though there is no wind turbine installed. Grid cells without wind turbines are shaded gray.

In all three case studies in Figure 7, there is a clear separation between areas with very low conditional probabilities and areas with higher conditional probabilities to experience UL. Areas of increased conditional probability of UL often include identified lightning flashes in that hour over the study area, although high conditional probability areas are often larger than the actual affected regions.

On 11 February 2020, shown in panels c and d of Figure 7, the study domain is again in a strong westerly flow associated with locally enhanced convective precipitation, CAPE, and strong maximum larger‐scale vertical velocities (not shown here). On 17 February 2020, the study area is crossed by a cold front at higher altitudes (above 500 hPa). Despite the different meteorological situation, the conditions are similar to the other case studies, showing elevated values in the three driver variables that strongly influence the conditional probability.

**Figure 8:**
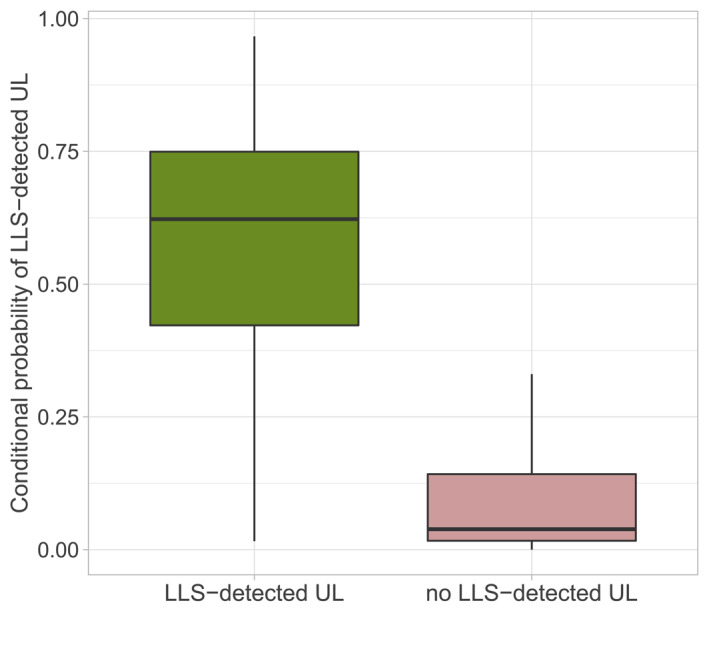
Left boxplot: computed conditional probability by tower‐trained random forest models for all Upward lightning (UL) situations on days with UL on at least one wind turbine detected by lightning location systems (LLS) within a specific grid cell. Right boxplot: conditional probability for no‐UL situations on days without UL detected by LLS within a specific grid cell.

Figure 8 summarizes the conditional probabilities comparing all days with UL at wind turbines (left boxplot) and all days without UL at wind turbines within the considered time period and the specific grid cells. As the case studies have illustrated, the random forest models trained at the two towers can clearly identify higher conditional probabilities (median: 0.62), when UL was observed within the considered hour and grid cell compared to lower conditional probabilities (median: 0.04), when no UL was observed. Conditional probability values close to 1 are not expected for a spatio‐temporal diagnosis, since the models must compute the probability of UL not only temporally, but also spatially for each grid cell.

Because it is the ability to discriminate between lower and higher conditional probabilities, rather than the absolute value of the conditional probability, the results support a generalized approach to risk assessment of UL on wind turbines.

### 4.2 Risk Assessment of UL at Wind Turbines

Identifying areas of increased probability of UL due to larger‐scale meteorological conditions is a valuable step in assessing the overall risk of lightning to tall structures >100 m, where wind turbines are most vulnerable. The previous analyses clearly show qualitatively and quantitatively that the tower‐trained random forest models distinguish between lower and higher conditional probabilities of UL at wind turbines. Further, observed lightning flashes at wind turbines frequently coincide with the areas of increased conditional probability computed by the random forest models. The following analysis considers the entire considered time period. Not only the models for LLS‐detected UL shall provide a risk assessment, but now the random forests for LLS‐detected + LLS‐undetected UL are applied to the study area and the considered time period.

The considered study period including the transition seasons and winter from 2018 to 2020 counts a total of 185 event days with 1,027 single flash hours and 18,602 single flash events. These numbers are intended as a measure to verify the resulting conditional probabilities from the random forest models. Note that these numbers are the lower bound of the number of flashes that actually occurred. Taking into account the uncertainty of manual identification of flashes at wind turbines as well as the uncertainty of UL detection by the LLS, a significantly higher number of actual lightning flashes at wind turbines can be expected. Furthermore, this verification approach only considers lightning at wind turbines and neglects all other tall structures such as radio towers in the study area that could be affected by UL. In the following, all days both with and without UL at wind turbines within the considered study period are taken as new data for the random forest models to compute the conditional probabilities on an hourly basis.

The goal is to identify regions that, according to the random forest models, have a higher risk of UL compared to other regions. This is done by counting the number of hours in each ERA5 grid cell (0.25° × 0.25°) that exceed the relatively high conditional probability threshold of 0.5.

**Figure 9:**
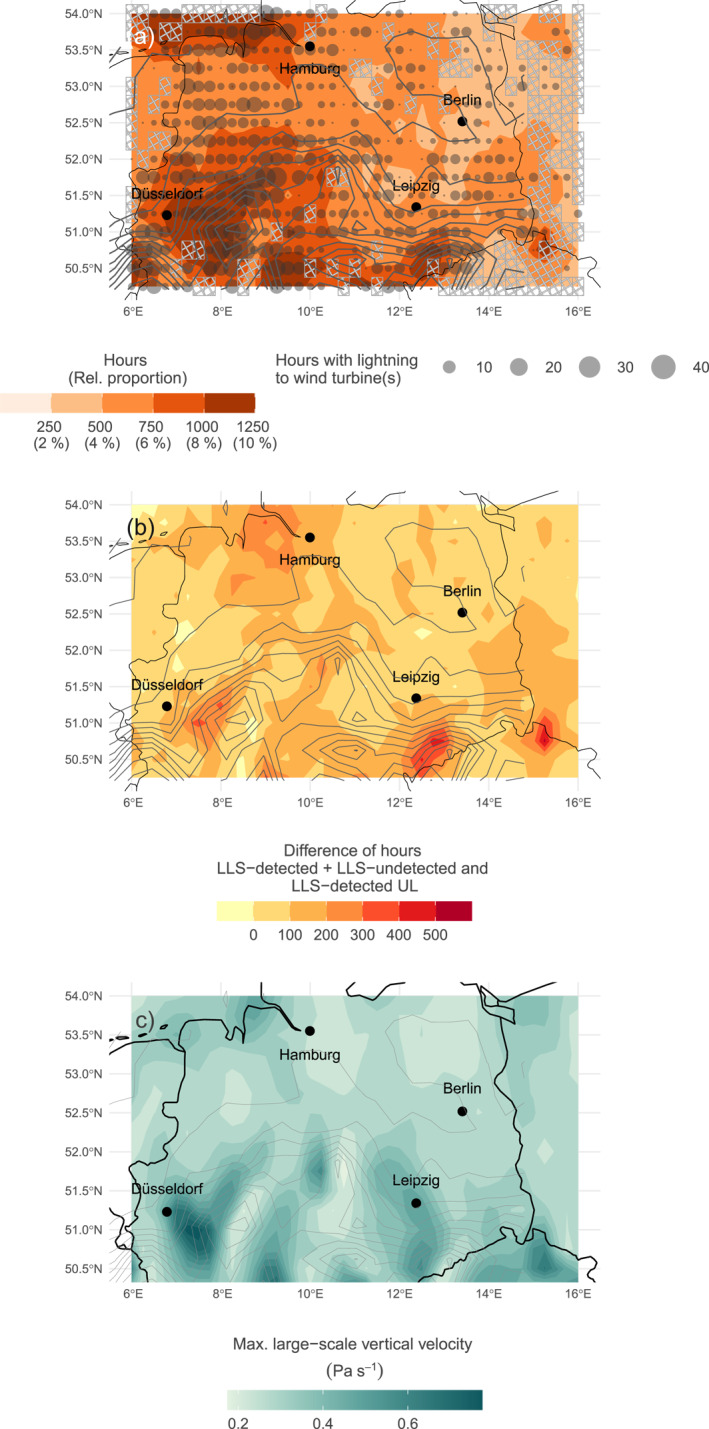
Panel (a) potential map for Upward lightning (UL) in the colder season (ONDJFMA) from 2018 to 2020 based on Gaisberg data (LLS‐detected + LLS‐undetected UL). Orange colors are median of hours per grid cell exceeding conditional probabilities of 0.5 according to 100 random forests. Transparent circles indicate the number of hours with UL at wind turbines detected by EUCLID, coded by size. Crosshatched areas indicate grid cells without wind turbines. Solid lines in all three panels show the topography (NASA Jet Propulsion Laboratory, California Institute of Technology, 2023). Relative proportion of in total 12,480 hr are given as reference. Panel (b) difference in hours between the results of the models based on Gaisberg data including (LLS‐detected + LLS‐undetected UL) and Gaisberg and Säntis data combined with only LLS‐detected UL. Panel (c) median of the maximum larger‐scale vertical velocity during EUCLID‐detected UL flashes.

Figure 9a based on LLS‐detected + LLS‐undetected UL illustrates that there are regions in the study area that have a higher risk of experiencing UL more frequently than other regions. The southern, north‐ and southwestern parts of the study area have a higher risk of LLS‐detected + LLS‐undetected UL.

Figure 9b, showing the difference between results based on LLS‐detected + LLS‐undetected UL and LLS‐detected UL only, illustrates that more flashes are expected when the LLS‐undetectable UL flash type is added. The pattern of areas with increased risk of experiencing UL is similar, although some of the more frequently affected areas are enlarged (see also Figure S3 in Supporting Information S1). This suggests that there are similar mechanisms resulting from large‐scale meteorology that lead to the LLS‐detected UL or LLS‐undetected UL flash types. A comparison of the affected regions with the actual lightning flashes at wind turbines extracted from the LLS EUCLID (circles, coded by size) suggests that in some areas a higher risk coincides more often with a higher number of hours with UL at wind turbines than in other areas. Near the coast and in the southern and southwestern part of the study area, the risk is most pronounced. Further northwest, however, the risk is lower, even though a relatively large number of hours with lightning flashes at wind turbines were measured, as indicated by the densely packed large circles. While the red colored areas reflect the risk of UL at wind turbines according to the prevailing larger‐scale meteorology alone, the LLS observations (circles) may be the result of various factors. These could be factors other than large‐scale meteorological conditions, which increase the risk of UL at wind turbines in these areas (Section 4.2, open issues). Toward the northeast of the study area, a lower number of observations, indicated by small circles with larger distances, often coincides with a lower risk of UL according to the larger‐scale meteorological setting.

Interestingly, the areas with higher probabilities of UL in Figure 9 often roughly coincide with regions close to elevated topography represented by the contour lines. Because random forests are not physically based but purely data‐driven models, no physical conclusions can be drawn from the random forest results alone. However, possible explanations include an increased effective height of the wind turbine (e.g., Shindo, 2018) or increased chances for thunderstorm formation due to orographic uplift. Furthermore the preferred wind direction from the western sector during LLS‐detected UL (see Figure S2 in Supporting Information S1) may lead to a vertical deflection of the near‐surface horizontal wind and to strong larger‐scale vertical velocities (Figure 9c). Stronger vertical velocities resulting from a sudden deceleration of the near‐surface horizontal wind when it blows from the ocean to land, or sea breezes, may also explain the higher probabilities in the northwesternmost, ocean‐covered part of the domain (Kirshbaum et al., 2018).

However, the slightly higher terrain in the southeastern part of the study area does not appear to significantly increase the risk of LLS‐detected UL. This may indicate that the terrain in the western part of the study area provides shelter from westerly winds, resulting in weaker wind speeds and hence weaker vertical velocities. Therefore, elevated terrain alone does not always increase the risk of UL at wind turbines, but it is a combination of topographic orientation and the flow impinging on elevated terrain.

The transfer of the random forest models trained with larger‐scale meteorological data from direct tower measurements to a larger region and its independent verification on wind turbines shows the potential of our approach to produce regionally varying risk maps, which in turn could lead to regionally varying (voluntary or enforced) lightning protection standards for wind turbines.

#### 4.2.1 Open Issues

This study has demonstrated a new approach to assessing the risk of UL at wind turbines using data from specially instrumented towers. However, there are some open issues that need to be discussed.

This study assesses the risk of UL based only on large‐scale meteorological variables. This does not mean that other factors do not influence the occurrence of UL at wind turbines. For example, meteorological variables at the cloud scale or even the physical characteristics of the structure, such as its height and shape, or the rotation of the wind turbine blades (Montanyà et al., 2014), may also play a role. Another important aspect that may increase the risk of UL at wind turbines is that wind farms with a large number of wind turbines may themselves increase the electric field sufficiently and lead to lightning “hotspots” (Soula et al., 2019).

The Gaisberg and Säntis Towers are located in different geographical locations with potentially different climatological settings. Further, they are located in complex terrain, which increases their effective height beyond their own height (Zhou et al., 2010). This may make them more susceptible to UL than wind turbines in flat terrain. Moreover, the effective height may be important for determining whether UL or DL occurs at the wind turbine. Unfortunately, these questions cannot be resolved with data from the EUCLID LLS used in this study since it does not distinguish between UL and DL. Therefore, it is not possible to conclude, whether and how the effective height affects the risk of UL at wind turbines. Additionally, the results of this study may not be generalizable to other seasons, such as the warmer season.

## 5 Conclusions

Upward lightning (UL) that strikes tall structures such as wind turbines is rare but can be critically destructive. Direct measurements of UL at a specially instrumented tower suggest that less than 50% of UL flashes can be detected by most LLS because they are unable to detect UL with only an ICC. Furthermore, UL is considered to be the main lightning type in the colder season.

The current international lightning protection standard (IEC 61400‐24, 2019) underestimates the risk of UL at wind turbines, as it does not sufficiently account for UL that is not detectable by LLS (LLS‐undetected UL) and for UL in the cold season. This study uses rare direct UL measurements at two specially instrumented Towers (Gaisberg Tower in Austria, Säntis Tower in Switzerland) with larger‐scale meteorological data in a machine learning model to estimate the risk of UL, including LLS‐undetected UL, on wind turbines in a study area in northern and central Germany.

The results show that UL can be reliably identified by the tower‐trained random forest models at the Gaisberg and Säntis towers. The larger‐scale meteorological drivers are large amounts of (convective) precipitation, strong larger‐scale vertical velocities, and slightly elevated CAPE. Furthermore, the vertical extent of the clouds and an increased amount of ice crystals and solid hydrometeors are important variables. Variables related to cloud physics and the wind field are stronger than the seasonal average and even stronger compared to situations with common DL in the vicinity of the Gaisberg Tower.

Extending the random forests to a larger domain shows that they can discriminate between regions with higher and lower probabilities, which often matches the observed lightning strikes at wind turbines. Extending the models trained on the Gaisberg tower to include LLS‐undetected UL flashes shows that areas with a higher risk of experiencing UL are expected to experience UL even more frequently.

The western and southern parts of the domain in northwestern Germany, close to elevated topography, and the coastal region in the northwesternmost part, are most at risk for UL at wind turbines, probably as a result of an interplay between the prevailing large‐scale wind field, which preferentially approaches the topography from the western sector.

This study demonstrates that direct UL measurements at two instrumented towers can be reliably modeled from larger‐scale meteorological conditions in a machine learning model (random forest). The study also proposes a novel way to justify the transfer of this model to a larger region using LLS‐detected UL data at wind turbine sites. As a result, regionally detailed risk maps of UL at wind turbines based on the larger‐scale meteorological setting can be produced.

## Conflict of Interest

The authors declare no conflicts of interest relevant to this study.

## Supporting information

Supporting Information S1

## Data Availability

ERA5 data are freely available at the Copernicus Climate Change Service (C3S) Climate Data Store (Hersbach et al., 2020). The results contain modified Copernicus Climate Change Service information (2020). Neither the European Commission nor ECMWF is responsible any use that may be made of the Copernicus information or data it contains. EUCLID data and ground truth lightning current measurements from the Gaisberg Tower are available only on request. For more details contact Wolfgang Schulz or Siemens BLIDS. All calculations as well as setting up the final data sets, modeling and the diagnosis were performed using R (R Core Team, 2021), using packages netCDF4 (Pierce, 2019), partykit (Hothorn & Zeileis, 2015), ggplot2 package (Wickham, 2016). Retrieving the raw data and deriving further variables from ERA5 required using Python3 (Van Rossum & Drake, 2009) and cdo (Schulzweida, 2019). OpenStreetMap contributors (2020) was used to extract wind turbine locations within the study domain.
