# Supplementary material for: Upward Lightning at Wind Turbines: Risk Assessment From Larger‐Scale Meteorology
Source: J Geophys Res Atmos. 2023 Dec 29;129(1):e2023JD039505. doi: 10.1029/2023JD039505 (PMC10909426; doi:10.1029/2023JD039505)
Supplement: Supplementary file 1 — Supporting Information S1 [file JGRD-129-e2023JD039505-s001.pdf]

Supporting Information for "Upward lightning at wind turbines: Risk assessment from larger-scale meteorology"

Isabell Stucke[1,2], Deborah Morgenstern[1,2], Gerhard Diendorfer[3], Georg J. Mayr[2], Hannes Pichler[3], Wolfgang Schulz[3], Thorsten Simon[4], Achim Zeileis[1]

[1]Institute of Statistics, University of Innsbruck, Austria, Innsbruck

[2]Institute of Atmospheric and Cryospheric Sciences, University of Innsbruck, Austria, Innsbruck

[3]OVE Service GmbH, Dept. ALDIS (Austrian Lightning Detection & Information System), Austria, Vienna

[4]Department of Mathematics, University of Innsbruck, Austria, Innsbruck

Contents of this file

1. Text Sections 1–3
2. Figure S1
3. Figure S2
4. Figure S3
5. Table S1

Introduction This supporting information file consists of five parts: text sections 1 to 3, three figures and one table. The text sections give information to the three figures.

Corresponding author: I. Stucke, Institute of Statistics University of Innsbruck, Innsbruck, Universitätsstrasse 15, 6020, Austria. (isabell.stucke@uibk.ac.at)

The first figure shows the seasonal flash density for the chosen study domain. The second figure shows the median of three different variables during LLS-detected UL: convective precipitation, CAPE and wind direction at 10 m. The third figure illustrates the results of an additional analysis using a different threshold to define regions with increased risk of UL at wind turbines. The final table lists all the variables included in the random forest models.

0.1. Seasonal flash density of the study domain

In the introduction of the article, the authors emphasize that the annual lightning activity is dominated by summer lightning activity. Figure S1 shows the seasonal total flash density for winter (DJF), summer (JJA), spring (MAM) and autumn (SON) and justifies this statement.

0.2. Convective precipitation, CAPE and wind direction at 10m during EUCLID-detected UL

Figure 9 c in the manuscript shows the median maximum larger-scale vertical velocity during EUCLID-detected UL. Two other relevant variables are convective precipitation and CAPE according to the variable importance analysis. The patterns of all three variables during EUCLID-detected UL look very similar to the patterns with regions showing the highest risk of UL at wind turbines. Panel c of Figure S2 shows that the preferred wind direction is west during LLS-detected UL.

0.3. Risk assessment of UL at wind turbines using a higher probability threshold

In Sect. 4.2 the model results for the risk assessment of LLS-detected + LLS-undetected UL are presented in the way that hours are counted exceeding a conditional probability

of 0.5. Figure S3 shows the risk assessment using a higher probability threshold, namely 0.8. The number of hours exceeding this threshold is lower by about a factor of two in comparison to a probability threshold of 0.5. However, the regional pattern is still similar with maxima West/South-West of the study domain.

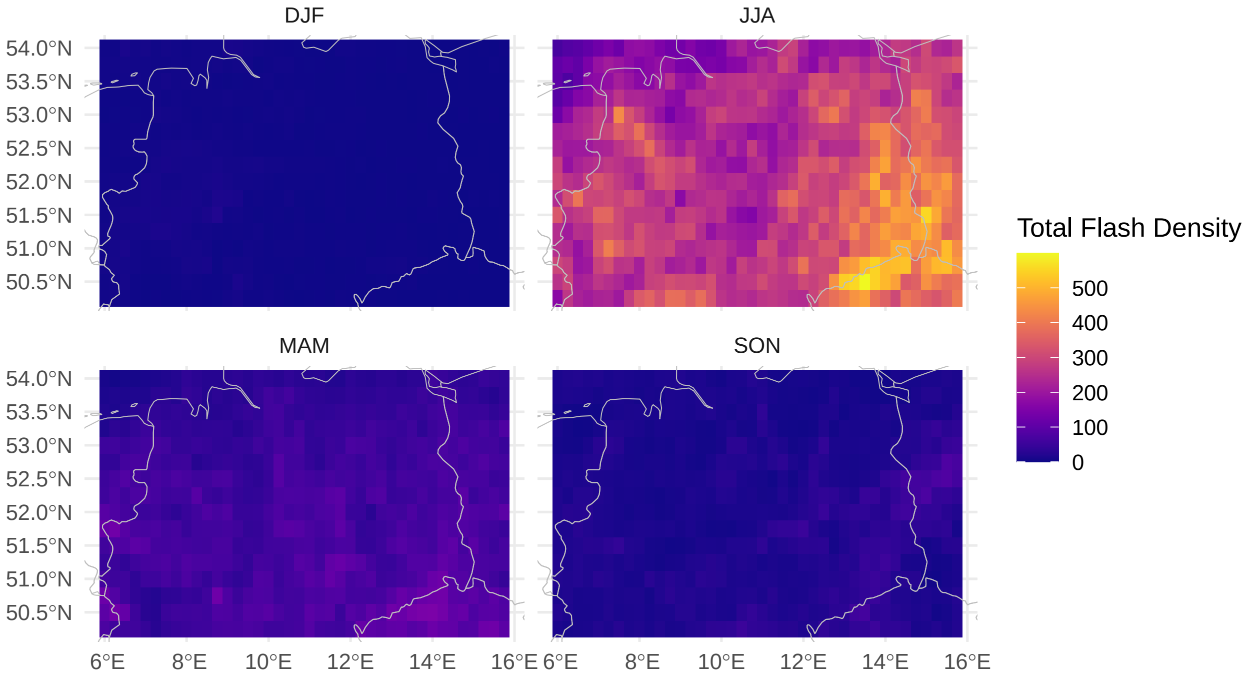

Figure S1. Analysis of total flash density in study domain per season and per ERA5 gridcell (ca. 31 km x 31 km). Data are based on EUCLID (2010 to 2020).

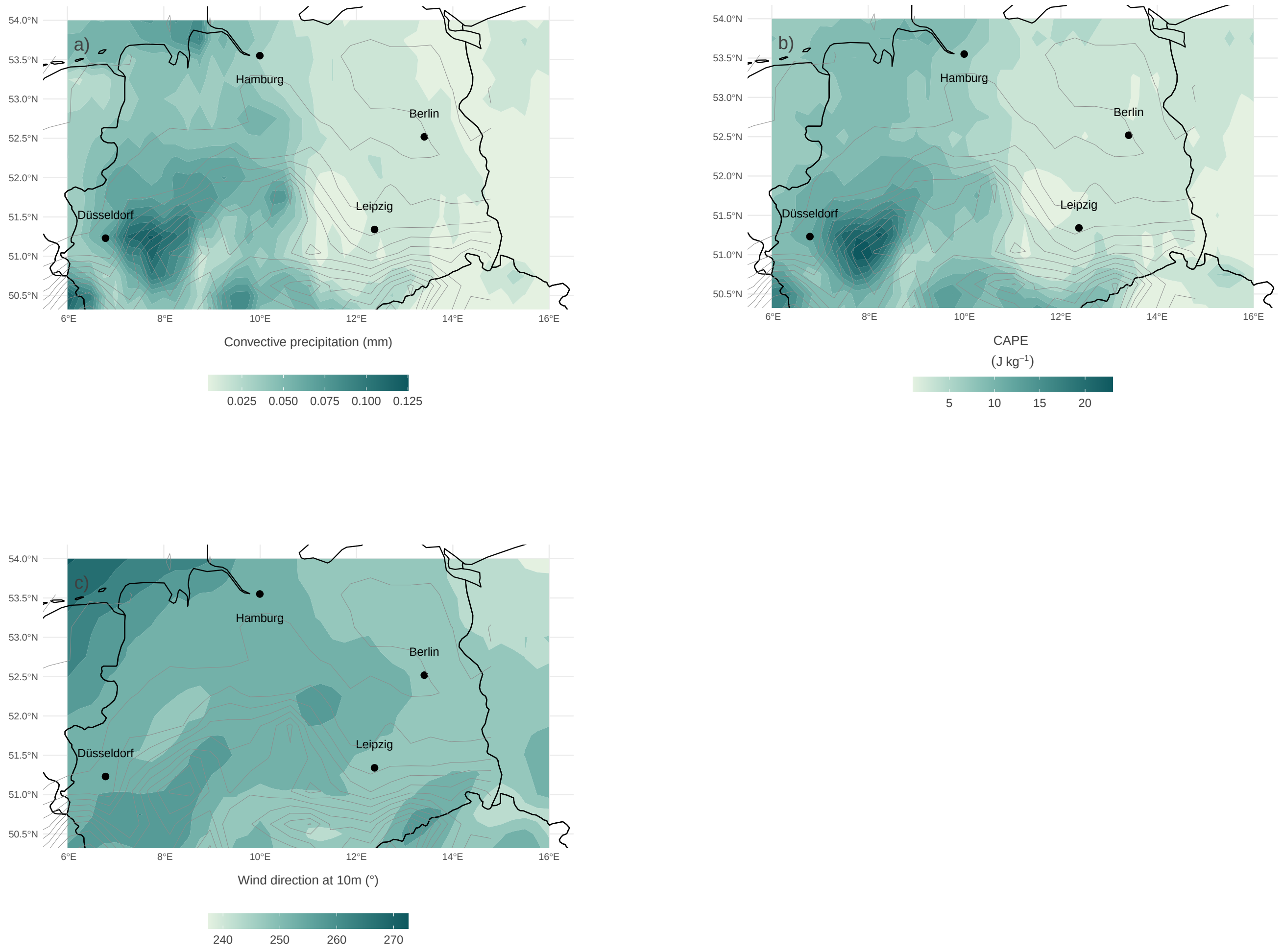

Figure S2. Panel a: median of convective precipitation during EUCLID-detected UL events. Panel b: median of CAPE during EUCLID-detected UL events. Panel c: median of the wind direction at 10 m during EUCLID-detected UL events.

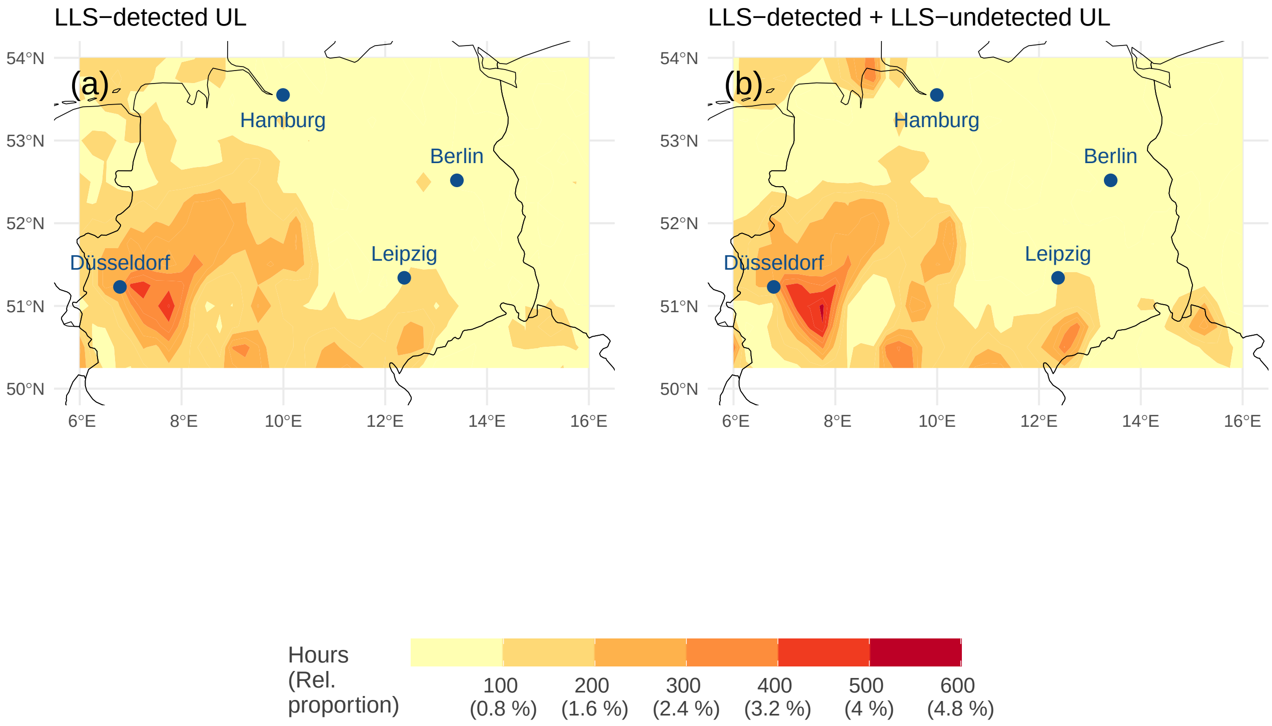

Figure S3. Panels (a) and (b): maps for the potential of UL in the colder season (OND-JFMA) from 2018 to 2020. Orange colors are median of hours per grid cell exceeding conditional probabilities of 0.8 according to 100 random forest models. Panel (a) shows results according to models based on Gaisberg and Säntis data combined. Panel (b) shows results according to models based on Gaisberg data also including the LLS-undetected UL. Relative proportions of in total 12480 hours are given as reference.

Table S1. Table of large-scale variables taken from ERA5 and variables derived from ERA5. The derived variables (indicated in italics) are suggested to be potentially important in the charging process of a thundercloud or for the development of convection.

**Large-scale variables**	**Unit**
cloud base height above ground	m agl
convective precipitation (rain + snow)	m
large scale precipitation	m
cloud size	m
maximum precipitation rate (rain + snow)	kg m^{-2} s^{-1}
ice crystals (total column, tciw)	kg m^{-2}
Solid hydrometeors (total column, tcsw)	kg m^{-2}
supercooled liquid water (total column, tcslw)	kg m^{-2}
water vapor (total column)	kg m^{-2}
vertical integral of divergence of cloud frozen water flux	kg m^{-2} s^{-1}
vertical transport of liquids around -10 *C*	kg Pa s^{-1}
ice crystals (-10 *C -* -20 *C)*	kg m^{-2}
ice crystals (-20 *C -* -40 *C)*	kg m^{-2}

cloud water droplets (−10 C - −20 C)	kg m^{-2}
solid hydrometeors (−10 C - −20 C)	kg m^{-2}
solid hydrometeors (−20 C - −40 C)	kg m^{-2}
solids (cswc + ciwc) around −10 C	kg m^{-2}
liquids (clwc + crwc) around −10 C	kg m^{-2}
2 m dew point temperature	K
mean vertically integrated moisture convergence	kg m^{-2} s^{-1}
water vapor (−10 C - −20 C)	kg m^{-2}
boundary layer height	m
surface latent heat flux	J m^{-2}
surface sensible heat flux	J m^{-2}
downward surface solar radiation	J m^{-2}
convective available potential energy	J kg^{-1}
convective inhibition present	binary
mean sea level pressure	Pa
height of −10 C isotherm	m agl
boundary layer dissipation	J m^{-2}

Maximum vertical updraft velocity	Pa s^{-1}
total cloud shear	m s^{-1}
wind speed at 10 m	m s^{-1}
wind direction at 10 m	$^\circ$
shear between 10 m and cloud base	m s^{-1}
